# Characterization and Comparative Transcriptomic Analysis of Skeletal Muscle in Pekin Duck at Different Growth Stages Using RNA-Seq

**DOI:** 10.3390/ani11030834

**Published:** 2021-03-16

**Authors:** Zhigang Hu, Junting Cao, Liyan Ge, Jianqin Zhang, Huilin Zhang, Xiaolin Liu

**Affiliations:** College of Animal Science and Technology, Northwest A&F University, Xianyang 712100, China; huzg2017060@163.com (Z.H.); fightingcaoting@nwafu.edu.cn (J.C.); a13149600612@163.com (L.G.); zhangjianqin@nwafu.edu.cn (J.Z.); zhhl7461@nwsuaf.edu.cn (H.Z.)

**Keywords:** skeletal muscle, transcriptome, DEG, pathway, Pekin duck

## Abstract

**Simple Summary:**

Skeletal muscle is an important tissue and its development is strictly regulated by genes. In this study, in order to understand the muscle-related gene expression in Pekin duck, RNA-seq was performed to analyze and compare skeletal muscle at different growth stages. Alternative splicing, single nucleotide polymorphisms and insertion–deletions were detected, and 299 novel genes were discovered. *MYL4*, *IGF2BP1*, *CSRP3*, *SPP1*, *KLHL31*, *LAMB2*, *LAMA2*, *ITGB1* and *OPN* played crucial roles in skeletal muscle development. Oxidative phosphorylation, ECM-receptor interaction, focal adhesion, carbon metabolism, and biosynthesis of amino acids participated in the regulation of skeletal muscle development in Pekin duck. This study provides an important reference for revealing the developmental mechanisms of pectoral and leg muscles in duck.

**Abstract:**

Skeletal muscle, accounting for approximately 50% of body weight, is the largest and most important tissue. In this study, the gene expression profiles and pathways in skeletal muscle of Pekin duck were investigated and compared at embryonic day 17, 21, and 27 and postnatally at 6 months of age. An average of 49,555,936 reads in each sample was obtained from the transcriptome libraries. Over 70.0% of alternative splicing (AS) in each sample was mainly alternative 5′ first exon (transcription start site)—the first exon splicing (TSS) and alternative 3′ last exon (transcription terminal site)—the last exon splicing (TTS), indicating that TSS and TTS were the most common AS event in Pekin ducks, and these AS events were closely related to the regulation of muscle development at different growth stages. The results provided a valuable genomic resource for selective breeding and functional studies of genes. A total of 299 novel genes with ≥2 exons were obtained. There were 294 to 2806 differentially expressed genes (DEGs) in each pairwise comparison of Pekin duck. Notably, 90 DEGs in breast muscle and 9 DEGs in leg muscle were co-expressed at all developmental points. DEGs were validated by qPCR analysis, which confirmed the tendency of the expression. DEGs related to muscle development were involved in biological processes such as “endodermal cell differentiation”, “muscle cell cellular homeostasis”, “skeletal muscle tissue growth” and “skeletal muscle cell differentiation”, and were involved in pathways such as oxidative phosphorylation, ECM-receptor (extracellular matrix receptor) interaction, focal adhesion, carbon metabolism, and biosynthesis of amino acids. Some DEGs, including *MYL*4, *IGF2BP1*, *CSRP3*, *SPP1* and *KLHL31*, as well as *LAMB2*, *LAMA2*, *ITGB1* and *OPN*, played crucial roles in muscle growth and development. This study provides valuable information about the expression profile of mRNAs and pathways from duck skeletal muscle at different growth stages, and further functional study of these mRNAs and pathways could provide new ideas for studying the molecular networks of growth and development in duck skeletal muscle.

## 1. Introduction

Skeletal muscle, which accounts for about 40%−50% of the bodyweight, is an important moving, defensive and metabolic organ [1]. The yield of animal muscle determines in part the economic income of livestock and poultry farmers. Many studies have shown that the development of skeletal muscle is not only influenced by nutrition and environment, but also strictly regulated by many genes, non-coding RNA and transcription factors [2,3]. In birds, skeletal muscles grow and develop rapidly during the embryonic period, and the numbers of muscle fiber continue to increase. On the contrary, the postnatal muscle fiber of birds becomes hypertrophy, and the nucleus of myofiber accumulate constantly, but the numbers no longer change [4,5]. Some studies have found that Gaoyou duck and Jinding duck have different daily gains in breast and leg muscle during the incubation period [6]. Pekin duck is popular because of its fast growth and good meat quality. It can weigh more than 3 kg at the age of two months, almost 60 times its birth weight (50 g). Therefore, Pekin duck is an ideal research subject for skeletal muscle growth and development.

Transcriptomics is an important tool for studying gene expression, cell phenotype and function. By using transcriptome sequencing technology, the expression patterns of a large number of genes at the mRNA level were detected, and the gene expression differences under different physiological and pathological conditions or at different time periods were compared, which provided important information for studying and revealing the core mechanism that regulates cell life activities [7]. The ultimate goal of transcriptome analysis is to define and quantify the precise map of transcripts and genes expressed in a given sample [8,9,10]. At present, the technology of transcriptomics has been increasingly improved, and it was used to study, compare and analyze gene expression in different fields [11], such as disease, evolution, comparative genomic resources, growth and development, immunity, reproductive physiology and environmental impact on organisms. Previous studies have reported the transcriptome expression of breast or/and leg muscle of poultry at different growth stages [12,13]. However, comparative transcriptomic studies on skeletal muscle of Pekin duck at different growth stages have been scarce.

To better understand muscle-related gene expression and improve the current annotation of duck genome, RNA-seq was performed to analyze and compare skeletal muscle of Pekin duck at different growth stages using the Illumina system. The SNPs (single nucleotide polymorphisms), InDels (insertion–deletions) and AS were screened to be used as important molecular markers in the genetic breeding of ducks. The DEGs and pathways were analyzed to find the mechanisms involved in skeletal muscle development in ducks using bioinformatics analysis. These results provided basic data for future research on duck muscle development.

## 2. Material and Methods

### 2.1. Ethics Approval and Consent to Participate

Animal care, slaughter and experimental procedures were approved by the Institutional Animal Care and Institutional Ethic Committee of Northwest A&F University (ethic code: #0330/2019).

### 2.2. Animal and Tissue Collection

One hundred eggs of Pekin duck (P) were incubated according to routine procedures after disinfection. On d 17 (E17), d 21 (E21) and d 27 (E27) of incubation, eight eggs (either male or female) were randomly selected to separate breast (B) and leg muscle (L) for DNA and RNA extraction. Because they are used to lay eggs, the majority of ducks on duck farms are female, and ducks of the same sex can avoid the error of sequencing data. The DNA of duck muscle was amplified by sex identification primers (gCHD, F: 5′-TGCAGAAGCAATATTACAAGT-3′; R: 5′-AATTCATTATCATCTGGTGG-3′) [14], and three female embryos were selected as the research object in each period (one band with 467 bp in male and two bands with 326 bp and 467 bp in female in agarose gel electrophoresis). In addition, 6-month-old female ducks (M6), raised under the same environmental conditions and free access to feed and water (Table S1), were slaughtered quickly to collect breast and leg muscle. The samples of breast and leg muscle (such as B1 and L1) were collected from the same bird, and the fresh tissues were immediately frozen in liquid nitrogen and then stored at −80 °C for preservation until use.

### 2.3. RNA Isolation, Library Preparation and Sequencing

According to the manufacturer’s instructions, the total RNA was extracted from breast and leg muscle at different growth stages using QIAzol Lysis Reagent (QIAGEN, Berlin, Germany). The concentration and integrity of RNA were determined using NanoDrop 2000 (Thermo, Waltham, MA, USA) and Bioanalyzer 2100 system (Agilent Technologies, San Jose, CA, USA), respectively. RNA purity was verified by agarose gel electrophoresis.

One single RNA-seq library was constructed for each RNA sample. The libraries for sequencing were constructed using the TruSeq PE Cluster Kit v4-cBot-HS (Illumia, San Diego, CA, USA) according to the manufacturer’s recommendations. Clusters of individual indexed samples were created using the cBot Cluster Generation System of Illumina following the manufacturer’s instructions. After cluster generation, the library preparations were sequenced on the Illumina platform (Illumina HiSeq X Ten platform, San Diego, CA, USA), with 150 bp pair-end reads generated.

### 2.4. Quality Control and Comparative Analysis

The high-quality clean data (filtered reads) were obtained by removing reads containing adapter or poly-N and low-quality reads from the raw data. In addition, Q30, GC content, and sequence duplication level of the clean data were calculated. All subsequent analyses were performed using high-quality clean data.

The clean reads were mapped to the Anas platyrhynchos (AP) genome sequence (https://www.ncbi.nlm.nih.gov/genome/?term=DUCK, accessed on 27 December 2019) and annotated transcripts (https://www.ncbi.nlm.nih.gov/assembly/GCF_003850225.1, accessed on 27 December 2019). Based on the AP genome, the perfect match reads or one mismatch reads were further analyzed and annotated. Then the HISAT 2 tool (http://ccb.jhu.edu/software/hisat2/index.shtml, accessed on 27 December 2019) was used to draw a duck genome map.

### 2.5. SNP/InDel Analysis and Prediction of AS

Based on the results of the HISAT 2 comparison between the reads of each sample and the AP genome sequence, the GATK software (https://software.broadinstitute.org/gatk/, accessed on 30 December 2019) was used to identify single-base mismatches and potential SNP sites [15]. It was determined whether these SNP sites affect the gene expression level or the type of protein product. Furthermore, the GATK can also be used to detect the InDels of each sample. To identify genomic variation from RNA-seq data, the highest possible accuracy of SNP calling needed to be achieved [16]. In this study, the raw vcffiles were filtered with GATK standard filter method and other parameters (clusterWindowSize: 10; MQ0 ≥ 4 and (MQ0/(1.0×DP)) > 0.1; QUAL < 10; QUAL < 30.0 or QD < 5.0 or HRun > 5) to minimize the rate of false-positive calls, and only SNPs with distance >5 were retained.

According to the position of mutation site and the gene location information in the AP genome, the SnpEff software was used to determine the region where mutation site occurs in the genome (intergenic region, gene region or CDS region, etc.), as well as the impact of mutation (synonymous and non-synonymous mutations, etc.). The StringTie software (https://ccb.jhu.edu/software/stringtie/index.shtml, accessed on 30 December 2019) was used to splice the comparison results of the HISAT 2, and the types of alternative splicing (AS) and corresponding expressions of each sample were obtained using the ASprofile software (http://ccb.jhu.edu/software/ASprofile/, ASprofile, The Center for Computational Biology at Johns Hopkins University, WA, USA). To better understand the location and function of AS in genes from duck skeletal muscle, and identify the types of AS mechanisms being used by genes expressed, twelve known types of AS were considered according to the structure of exons [17], including (a) TSS: Alternative 5′ first exon (transcription start site) the first exon splicing; (b) TTS: Alternative 3′ last exon (transcription terminal site) the last exon splicing; (c) SKIP: Skipped exon single exon skipping; (d) XSKIP: Approximate SKIP single exon skipping (fuzzy boundary); (e) MSKIP: Multi-exon SKIP multi-exon skipping; (f) XMSKIP: Approximate MSKIP multi-exon skipping (fuzzy boundary); (g) IR: Intron retention single intron retention; (h) XIR: Approximate IR single intron retention (fuzzy boundary); (i) MIR: Multi-IR multi-intron retention; (j) XMIR: Approximate MIR multi-intron retention (fuzzy boundary); (k) AE: Alternative exon ends (5′, 3′, or both); (l) XAE: Approximate AE variable 5′ or 3′ end (fuzzy boundary).

### 2.6. New Genes Analysis

Based on the AP genome sequence, the mapped reads were spliced by the StringTie software (https://ccb.jhu.edu/software/stringtie/index.shtml, The Center for Computational Biology at Johns Hopkins University, Baltimore, MD, USA), and then the spliced sequences were compared with the original genome annotation information to find the uncommented transcription area and explore the new transcripts and new genes of the species. Finally, the original genome annotation information was supplemented and improved.

The BLAST software (https://blast.ncbi.nlm.nih.gov/Blast.cgi, National Center for Biotechnology Information, Bethesda, MD, USA.) was used to compare the newly discovered genes with the NR (NCBI non-redundant protein sequences, ftp://ftp.ncbi.nih.gov/blast/db/, accessed on 30 December 2019), Swiss-Prot (a manually annotated and reviewed protein sequence database, http://www.uniprot.org/, accessed on 30 December 2019), GO (Gene Ontology, http://www.geneontology.org/), COG/KOG (Clusters of Orthologous Groups of proteins, http://www.ncbi.nlm.nih.gov/COG/; http://www.ncbi.nlm.nih.gov/KOG/, accessed on 30 December 2019), Pfam (Protein family, http://pfam.xfam.org/, accessed on 30 December 2019), and KEGG (Kyoto Encyclopedia of Genes and Genomes, https://www.genome.jp/kegg/ko.html, accessed on 30 December 2019) databases. The KOBAS 2.0 was used to obtain the KEGG Orthology results of the new genes. After predicting the amino acid sequence of new genes, the HMMER software (http://www.hmmer.org/, accessed on 30 December 2019, Howard Hughes Medical Institute, Chevy Chase, MD, USA) was used to compare with the Pfam database to get annotation information of new genes.

### 2.7. Quantification and Differential Expression Analysis

The FPKM (fragments per kilobase per transcript per million mapped reads) method was performed to quantify the expression of two expressed profiles, and the FPKM was calculated based on the length of gene and the read counts mapped to the AP genome. Formula was as follows:

$$FPKM=\frac{\mathrm{cDNA}\;\mathrm{Fragments}}{\mathrm{Mapped}\;\mathrm{Fragments}(\mathrm{Millions})\times \mathrm{Transcript}\;\mathrm{Length}(\mathrm{kb})}$$

The cDNA Fragments refer to the number of fragments compared to a transcript; Mapped Fragments (Millions) refer to the total number of fragments compared to a transcript, in 1 × 10^6^ units; Script Length (kb): the length of the transcript, in 1 × 10^3^ base units.

The DESeq2 software (http://www.bioconductor.org/packages/release/bioc/html/DESeq.html, accessed on 30 December 2019 DESeq2-1.28.1, Bioconductor, Buffalo, NY, USA) was used for differential expression analysis between sample groups to obtain a DEG set between two biological comparisons. The false discovery rate (FDR) method was used for hypothesis testing and multiple hypothesis testing to calibrate the significance level and eliminate the influence of random fluctuations and errors. Fold change ≥2 and FDR < 0.01 were used as the criteria for screening DEGs. Because differential expression analysis of transcriptome sequencing is an independent statistical hypothesis test on a large number of gene expression values, there will be false-positives problems. Therefore, the Benjamin–Hochberg correction method was used to correct the significant *p*-value of the original hypothesis test, and FDR was used as a key indicator for screening DEGs.

### 2.8. Analysis of GO Enrichment and KEGG Pathway Enrichment

Gene functions of duck (Anas platyrhynchos, https://www.ncbi.nlm.nih.gov/genome/?term=DUCK, accessed on 30 December 2019; https://www.ncbi.nlm.nih.gov/assembly/GCF_003850225.1, accessed on 28 December 2019) were annotated with the following databases, NR; Nt (NCBI non-redundant nucleotide sequences); Pfam; KOG/COG; Swiss-Prot; GO; KO (KEGG Ortholog database). GO enrichment of DEGs was analyzed by the GOseq R software package, which can adjust for gene length bias in DEGs. The KOBAS software was used to test the statistical enrichment of DEGs in KEGG pathway and the high-level functions of biological system were further understood.

### 2.9. Protein–Protein Interaction Network

Combining the results of differential expression analysis and the interaction pairs contained in the database, the protein–protein interaction (PPI) was constructed using the STRING database (http://string-db.org/, accessed on 28 December 2019). For the species not included in the database, the homologous protein was found using the BLAST software to sequence the target gene and the protein in the database. Then, the interaction network was constructed according to the interaction relationship of homologous proteins. The PPI of these DEGs was visualized by the Cytoscape software (http://www.cytoscape.org/, accessed on 28 December 2019).

### 2.10. Verification of Results by qPCR

Twelve DEGs were randomly selected from the gene expression data obtained by RNA sequencing in Pekin duck to verify their repeatability and reproducibility by qPCR. The first strand of cDNA from eight embryos (including three samples of transcriptome sequencing) in each age group was synthesized according to the manual of reverse transcription kit (abm, Richmond, Canada). Gene-specific primers were designed based on DEGs, and β-actin and GAPDH of duck were designed based on GenBank (accession number: NC_040060.1) by the Primer 5.0 software (Table 1), after the stability (average expression stability, M) of β-actin and GAPDH was tested using the geNorm software (https://genorm.cmgg.be/), and finally β-actin was used as a housekeeping gene (β-actin, M = 0.551; GAPDH, M = 0.722). Gene expression was performed in triplicate with 5 μL 2 × TransStart Tip Green qPCR SuperMix (Transgen, Beijing, China), 0.3 μL of each primer (10 μM), 0.8 μL cDNA (400 ng/μL) and 3.6 μL ddH_2_O using EcoRT48 (OSA, London, UK). The optimal reaction procedure included 95 °C for 30 s, followed by 40 cycles of 95 °C for 5 s, 60 °C for 30 s, then 95 °C for 15 s, 55 °C for 15 s, 95 °C for 15 s. The relative expression levels of DEGs were calculated by 2^−^^△△Ct^ method. Data were presented as mean ± SD. Differences between means were analyzed using one-way analysis of variance (ANOVA) followed by Duncan’s test and Tukey’s test. “*” was considered significant difference (*p* < 0.05); “**” was considered highly significant difference (*p* < 0.01).

## 3. Results

### 3.1. Evaluation of Transcriptome Profiles

In order to determine the potential candidate genes that may affect the development of skeletal muscle in Pekin duck, RNA-seq was used to investigate the gene expression profiles in breast and leg muscle at different growth stages. RNA was extracted from skeletal muscle in duck at growth stages of E17d, E21d, E27d and M6. The average RIN was 7.6 for all RNA samples, and the libraries were constructed and sequenced after all the samples passed the quality test (Table S2). After filtering the raw data, more than 21,134,566 clean reads were received in each sample, GC content was greater than 50.37%, and Q30 value was higher than 92.59%, indicating that the sequencing data had a good reproducibility. (Table 2).

A total of 1,189,342,460 reads (not paired-end reads) were obtained from the transcriptome libraries with an average of 49,555,936 reads in each sample (the numbers of reads ranging from 42,269,132 to 62,307,094). The number of reads mapped to the AP genome and the percentage of clean reads were not less than 31,111,384 and 62.39%, respectively. The number of uniquely mapped reads was 25,355,459 to 45,171,228, and the uniquely mapped rates were 55.34% to 73.80%. Of the total clean reads, at least 1,715,734 reads aligned to two or more locations in the AP genome, and the percentage of multiple mapped reads ranged from 3.45% to 20.24% (Table S3).

### 3.2. Annotation and Classification of SNP/InDel

**Figure 1:**
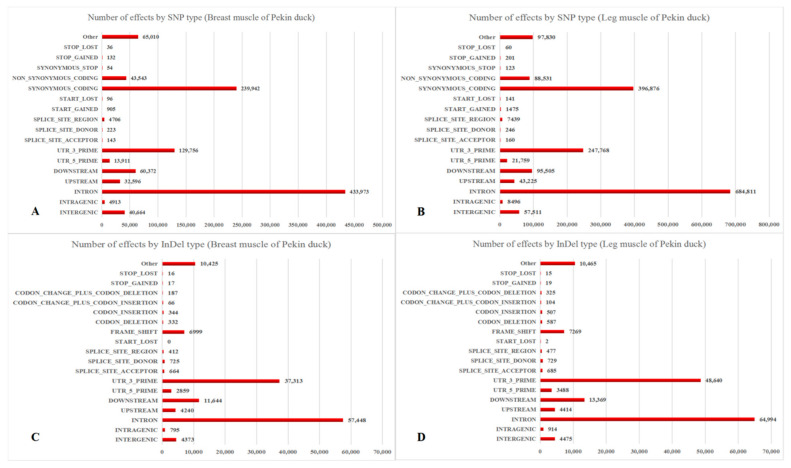
Annotation and classification of SNPs (single nucleotide polymorphisms) and InDels (insertion-deletions) in Pekin duck. (**A**) SNPs of breast muscle; (**B**) SNPs of leg muscle; (**C**) InDels of breast muscle; (**D**) InDels of leg muscle.

The number of SNP sites, the proportion of conversion/transversion type, and the ratio of heterozygous SNP sites in each sample were counted, as shown in Table S4. The SNP numbers were 64,295 to 427,493 in skeletal muscle of Pekin duck. The total numbers of SNP in the genic region were 57,351 to 392,907, and the total numbers of SNP between genes were 5486 to 34,586 in Pekin duck muscle. The annotation results of SNP and InDel were shown in Figure 1.

### 3.3. Prediction of Alternative Splicing

**Figure 2:**
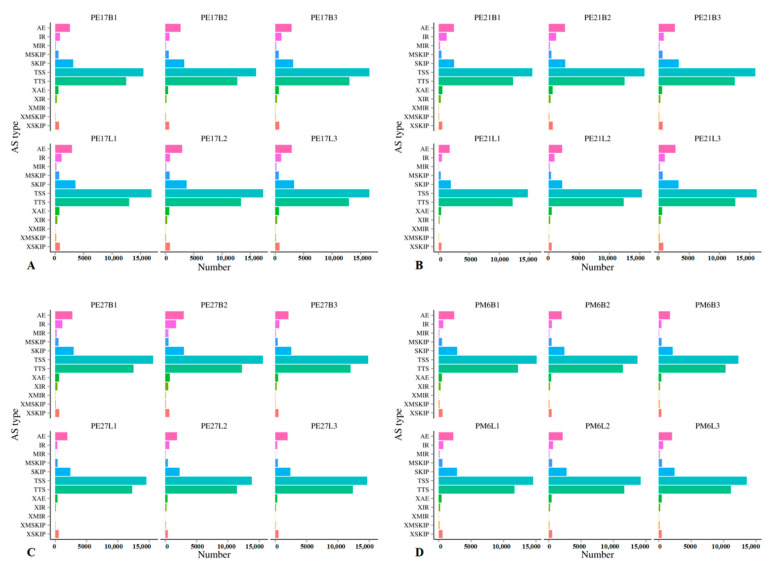
The predicted number of alternative splicing in Pekin ducks during different incubation stages. (**A**–**D**) represent the predicted number of alternative splicing in E17d, E21d, E27d and M6, respectively.

All 12 known types of AS were found in 24 libraries. The numbers of AS were 32,256 to 45,707 in breast muscle, and 35,841 to 47,889 in leg muscle at different time points (Table 3). As shown in Figure 2, the types of AS event in each sample were mainly concentrated in Alternative 5′ first exon (transcription start site)-the first exon splicing (TSS), Alternative 3′ last exon (transcription terminal site)-the last exon splicing (TTS), skipped exon single exon skipping (SKIP) and Alternative exon ends (5′, 3′, or both) (AE). Most of the AS genes produced two or more isoforms (Table S5). Notably, TSS and TTS accounted for more than 70% of the total, suggesting that they are the most common AS events in skeletal muscle of Pekin duck. The results provided a good reference for AS events that occur in duck muscle development.

### 3.4. New Genes Analysis

Based on the alignment of the sequencing data to the AP genome, a total of 299 novel genes with ≥2 exons were obtained. The number of novel genes annotated by various databases was summarized in Table 4.

### 3.5. Gene Functional Annotation and Classification

According to the AP genome sequence, mapped reads were spliced using the StringTie software, and these reads were compared with the original genome annotated information to find uncommented transcription regions, then the previous genome annotated information was complemented and improved. The new genes were compared with the databases of NR, Swiss-Prot, GO, COG, KOG, Pfam and KEGG using the BLAST software. The KEGG Orthology results of new genes were obtained by the KOBAS 2.0, and the amino acid sequences of novel genes were blasted against Pfam database by the HMMER tool to gain the annotation information (Table 5).

### 3.6. Analysis of Differentially Expressed Genes

**Figure 3:**
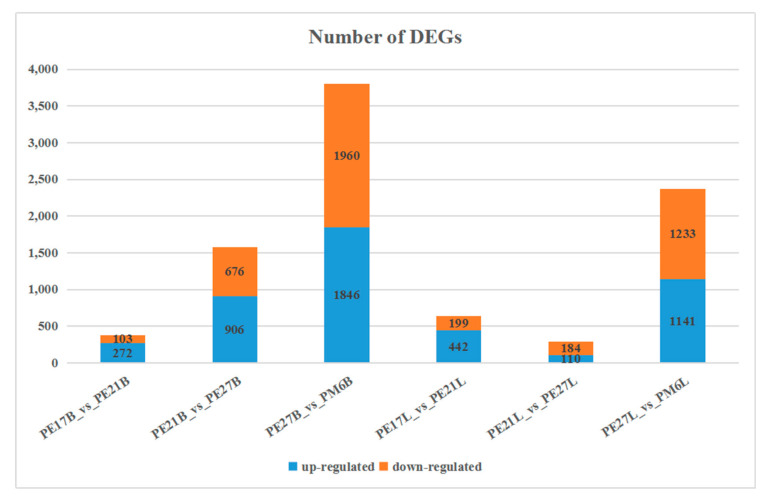
Number of DEGs during skeletal muscle development in Pekin duck.

**Figure 4:**
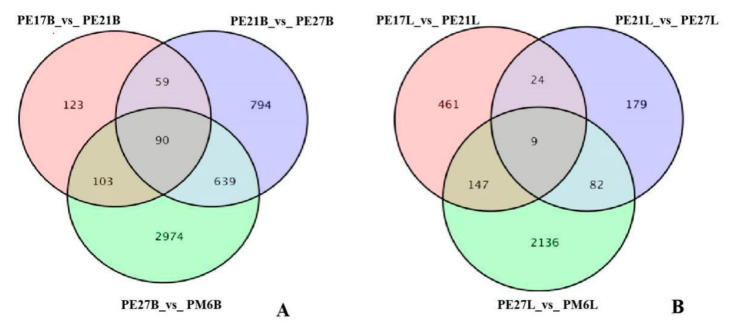
Venn diagram of differentially expressed genes among the three comparison groups. (**A**) The co-expressed DEGs in breast muscle of Pekin duck; (**B**) the co-expressed DEGs in leg muscle of Pekin duck.

In order for the number of fragments to truly reflect the transcript expression level, the number of mapped reads and transcript length in samples needed to be normalized. To quantify the basic genetic difference between breast and leg muscle of Pekin duck, DEGs in transcriptome were analyzed at different growth stages. A pairwise comparison was conducted using a t-test (Fold change ≥2 and FDR < 0.01) to identify DEGs between the two muscle tissues. In breast muscle of Pekin duck, 375 genes were significantly differentially expressed, including 272 up-regulated and 103 down-regulated genes in PE17B_vs_PE21B. Moreover, there were 1582 significantly expressed genes from PE21B_vs_PE27B, among which 906 were up-regulated genes and 676 were down-regulated genes. The number of DEGs detected from PE27B_vs_PM6B was 2806, the number of up-regulated genes were 1846 and the down-regulated genes were 1960. In leg muscle of Pekin duck, there were 641 DEGs from PE17L_vs_PE21L, including 442 up-regulated genes and 199 down-regulated genes. A total of 294 DEGs were found in PE21L_vs_PE27L, of which 110 were up-regulated genes and 184 were down-regulated genes. In addition, 2374 DEGs were discovered in PE27L_vs_PM6L, and 1141 DEGs were up-regulated genes and 1233 DEGs were down-regulated genes (Figure 3). Across all developmental time points, 90 DEGs in breast muscle and 9 DEGs in leg muscle were co-expressed (Figure 4).

### 3.7. GO Annotation and KEGG Pathway Analysis

**Figure 5:**
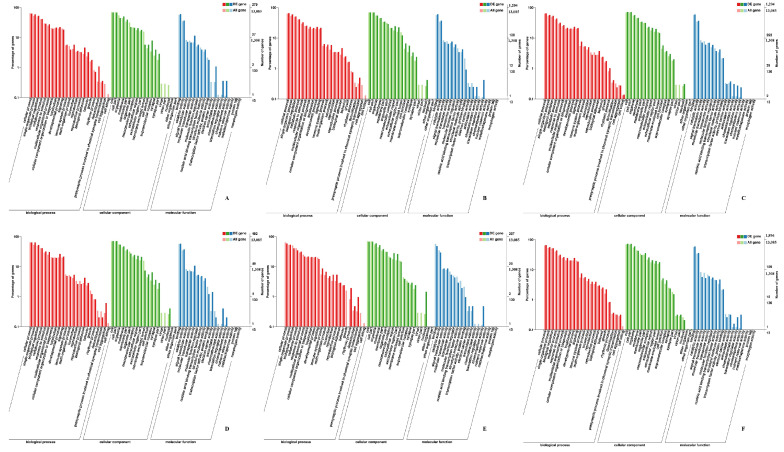
GO enrichment analysis of DEGs in Pekin duck. (**A**) PE17B_vs_PE21B; (**B**) PE21B_vs_PE27B; (**C**) PE27B_vs_PM6B; (**D**) PE17L_vs_PE21L; (**E**) PE21L_vs_PE27L; (**F**) PE27L_vs_PM6L. Note: The abscissa were GO terms, the ordinate on the left was percentage of genes in all genes annotated with GO, on the right was the number of genes.

To further determine the function of DEGs, GO and KEGG pathway analyses were performed to search for significantly overrepresented categories. GO terms were analyzed from three aspects: cell composition, molecular function and biological process. In skeletal muscle of Pekin duck, most DEGs were enriched in the category of cellular components related to muscle development, and “myosin complex”, “myofibril” and “proteinaceous extracellular matrix” were the most important subcategories (Table S6). For the molecular function category associated with muscle development, three most abundant subcategories were “extracellular matrix structural constituent”, “muscle alpha-actinin binding”, and “microtubule motor activity” (Table S7). As for the biological process category, most DEGs were assigned to “endodermal cell differentiation”, “muscle cell cellular homeostasis”, “skeletal muscle tissue growth” and “skeletal muscle cell differentiation” (Table S8), and the genes *ACTN1*, *FGF19* and *MAP6* that participated in skeletal muscle development were differentially expressed in this term. In addition, *OPN*, *GAS2*, *HTRA1*, *LAMB2*, *LAMA2*, *SERPINH1*, *ITGB1* and *GLI2*, which are responsible for muscle synthesis or metabolism, were highly enriched in the biological process (Figure 5).

**Figure 6:**
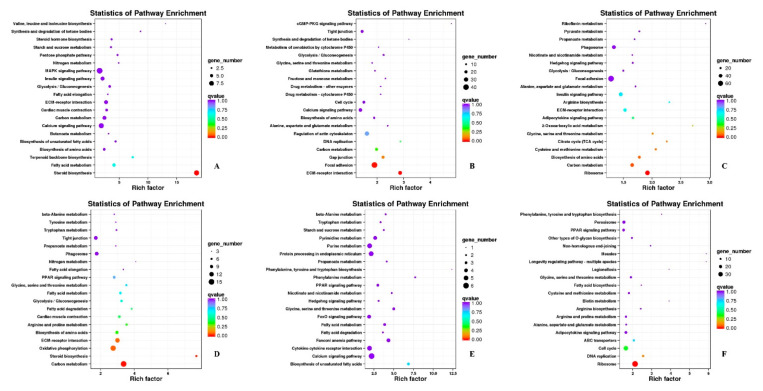
KEGG annotation of DEGs in Pekin duck. (**A**) PE17B_vs_PE21B; (**B**) PE21B_vs_PE27B; (**C**) PE27B_vs_PM6B; (**D**) PE17L_vs_PE21L; (**E**) PE21L_vs_PE27L; (**F**) PE27L_vs_PM6L. Note: Each circle represented a KEGG pathway, the name of which was shown on the left legend. Abscissa indicated enrichment factors, showing the proportion of (a) to (b), (a) was the ratio of differentially expressed genes in the pathway with all DEGs in all pathways, (b) was the ratio of genes in the pathway with all genes in all pathways. The bigger the Rich factor is, the more significant the pathway is. The color of circle represented q value which is adjusted *p* value by multiple hypothesis testing. Thus, the smaller the q value is, the more significant the pathway is; the circle size represented the number of differentially expressed genes annotated with the pathway, the bigger circle size is, the higher number of genes is.

DEGs were annotated to identify enriched pathways using the KOBAS (Figure 6). These pathways were significantly enriched, including oxidative phosphorylation, ECM-receptor (extracellular matrix receptor) interaction, focal adhesion, carbon metabolism and biosynthesis of amino acids. Four genes, *LAMB2*, *LAMA2*, *ITGB1* and *OPN*, were highly enriched in GO terms and signifcantly expressed in KEGG pathways to regulate the development of skeletal muscle.

### 3.8. Analysis of Protein–Protein Interaction Network

After analysis of GO and KEGG pathway, several DEGs played a core role in the PPI network, including *LAMB2*, *LAMA2*, *ITGB1*, *IGF2BP1*, *SPP1*, *KLHL31*, *CSRP3*, and *OPN*, indicating that these genes may play key roles in regulating muscle growth of ducks (Figures S1–S6).

### 3.9. Validation of RNA-Seq Results

**Figure 7:**
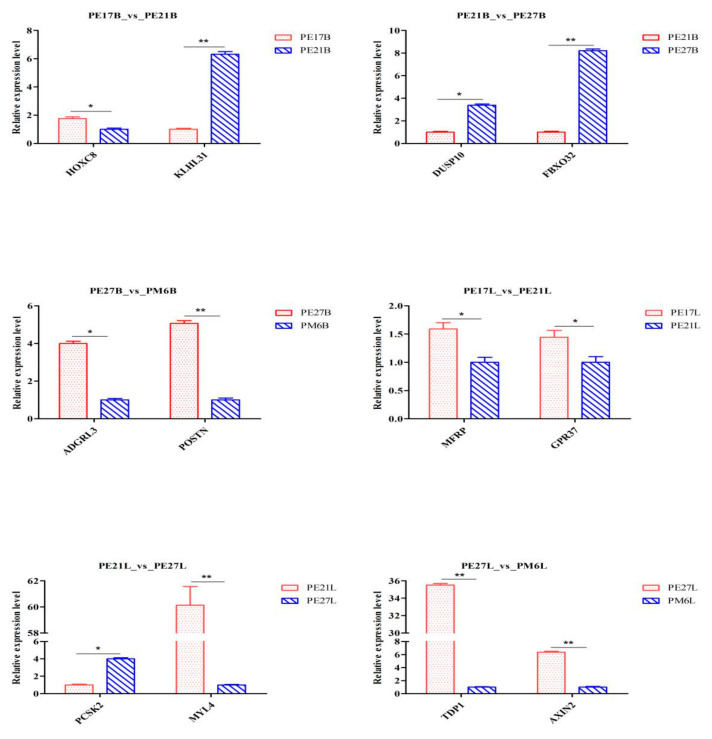
qPCR verification of DEGs. “*” was considered significant difference (*p* < 0.05); “**” was considered highly significant difference (*p* < 0.01).

In order to verify DEGs obtained in skeletal muscle of Pekin duck at different growth stages, twelve genes were randomly selected, and their expression levels were quantified by qPCR (Figure 7). The results indicated that the expression of genes had a similar down- or up-regulation trend.

## 4. Discussion

Development of skeletal muscle is a tightly regulated process that is critically modulated by genes and related signaling. In this study, the major DEGs and their expression pathways in skeletal muscle of Pekin duck were investigated using RNA-Seq technology and bioinformatic tools.

### 4.1. Analysis Summary of Sequencing Data

In this study, a total of 24 libraries in breast and leg muscles of Pekin ducks were established by high-throughput RNA sequencing, and the clean reads of each sample were obtained, which are very important to understand the gene expression patterns in the skeletal muscle development to improve the muscle production rate in poultry. A total of 299 novel annotated genes with two or more exons were obtained in Pekin duck, which will help to understand its function and improve the current annotation of duck genome. All the selected DEGs showed concordant expression patterns between the RNA-Seq and qPCR results. The sequencing data greatly improved the existing gene annotations.

### 4.2. Annotation and Classification of SNP/InDel in Skeletal Muscle Developmental Process

RNA-seq not only measures gene expression but also structural variations such as SNPs and InDels. The SNPs in the coding region could be divided into synonymous and non-synonymous, and the protein sequences are affected by the latter. SNPs have been applied as important molecular markers in animal genetics and breeding studies [18,19]. Lee, E.A. et al. found that the g.489 C > T and g.1264 C > A SNPs in *MYOD1* were meaningful markers to improve the lean meat production and meat quality of pigs [20]. It was also found that variation in intron 2 of *GH* (C172T) may be a molecular marker for superior growth and carcass traits in Cherry Valley duck, Muscovy duck and Jingjiang duck [21]. Many skeletal muscle SNPs were discovered using transcriptomic sequencing [22,23]. In this study, a range of 64,295 to 427,493 SNPs was found in skeletal muscle of Pekin duck. The total numbers of SNPs in the genic region were 57,351 to 392,907, and the total numbers of SNPs between genes were 5486 to 34,586 in Pekin duck muscle. Many discrepancies were found in the number of SNPs because the individual SNP mutations were affected by many factors, including genetics and the presence of SNPs in unknown/new transcripts, and many SNPs were non-synonymous in a biological population. Intergenic SNPs were important when considering the markers density and genome coverage using SNP marker, especially when these SNPs were used for linkage map construction [24]. The SNPs and InDels distribution among all genes from duck skeletal muscke were analyzed. The annotations of SNP were mainly “INTRON”, “SYNONYMOUS_CODING” and “UTR_3_PRIME”, and the annotations of InDel were mainly “INTRON”, “UTR_3_PRIME” and “DOWNSTREAM” in skeletal muscle of Pekin duck. The 3′ UTR region of gene was involved in the regulation of mRNA transcription, secondary structure, stability, localization and translation, and bound regulators like miRNAs and RNA-binding proteins [25]. In addition, mutation affected expression of downstream genes [26]. The SNPs and InDels from the “INTRON”, “SYNONYMOUS_CODING” may have smaller influence on final protein sequences, but the SNPs and InDels of “UTR_3_PRIME” and “DOWNSTREAM” may be important in gene regulation. Therefore, these SNPs and InDels may improve the current annotation of duck genome and become important molecular markers in duck genetic breeding.

### 4.3. Alternative Splicing Events in Skeletal Muscle Developmental Process

The high resolution of RNA-seq data allows for exploring not only gene expression but also AS events in animals. AS is ubiquitous in eukaryotes. It brings significant protein diversity and allows a gene to generate different mRNA transcripts, which are translated into different proteins. In animals, some studies highlighted the important role of AS in genes [27,28]. Notably, AS plays an important role in muscle development [29,30]. Some studies have explained how regulated AS of sarcomeric proteins in both flies and mammals can directly instruct the physiological and biophysical differences between fiber-types [31]. Chen, P.R. et al. found that exogenous expression of myostatin AS variant promoted fiber proliferation of leg muscle in Japanese quail [32]. Zhang, C.L. et al. found that there were the same rates of AS (alternative 3′ splicing site (A3SS), alternative 5′ splicing site (A5SS), intron retention (IR) and exon skipping (ES)), and accounted for more than one-third of all AS events in the the biceps brachii of Small-tailed Han sheep and Dorper sheep [33]. In this study, the four primary types of AS events, namely TSS, TTS, SKIP and AE were common in the skeletal muscle at all time points. Most of the AS genes, such as *MyoG*, *MYL4* and *IGF2BP1*, produced two or more isoforms. Several muscle related genes, such as *MEF2A*, *DYSF* and *MATR3*, were identified with more than 10 alternative transcripts. Over 70.0% of the AS events were TSS and TTS at all growth stages, which was consistent with previous studies on AS of pig skeletal muscle [34,35], indicating that TSS and TTS were the most common AS events in Pekin ducks, and these AS events were closely related to the regulation of muscle development at different growth stages. Although these two events may be located in the un-translated region (UTR) and may have smaller impact on the final protein sequences than IR, SKIP and AE types, the 5′ UTR and 3′ UTR, regulating the transcription and translation of mRNA, were essential in biological processes [36]. These results suggested that the identification of AS events will contribute to a better understanding of the regulatory mechanisms during the skeletal muscle myogenesis of Pekin duck.

### 4.4. DEGs Analyzed at All Time Points

The characterization of gene expression is a powerful method to identify the differences of transcriptional machinery between tissues and different states of cells. Pekin duck displayed significantly different muscle growth and development performance at different time points. To investigate potential genes and pathways that are involved in muscle growth and development, transcriptomic analysis was performed in skeletal muscle of Pekin duck. The expression of DEGs varies greatly at different developmental stages., and several genes were continuously up- or down-regulated in the whole development process, such as *MYL4*, *IGF2BP1*, *CSRP3*, *SPP1* and *KLHL31*. The essential growth differences of skeletal muscle in Pekin duck may be driven by these sustained DEGs.

Myosin light chain 4 (*MYL4*) gene, presenting at a high level in the initial stage of muscle development, is down regulated after birth and re-expressed during muscle regeneration [37]. Li, D.F. et al. found that *MYL4* was related to muscle fiber hypertrophy of yellow broilers by transcriptome sequencing [38]. Ye, M.S. et al. found that *MYL4* gene regulated and affected the development of muscle fiber through transcriptome sequencing [39]. Ciecierska, A. et al. indicated that *MYL4* was highly expressed in muscle cells of beef breeds [40]. In our study, *MYL4* may be an important regulator in the development of duck skeletal muscle.

It is well known that IGFs promote the growth and development of organisms. IGFBPs compete with cell surface receptors for free IGF1 and IGF2 and regulate the expression of IGFs in target cells [41]. Insulin-like growth factor 2 mRNA-binding protein 1 (IGF2BP1), a member of a conserved family of single-stranded RNA-binding proteins, is involved in regulating embryogenesis, cell migration, proliferation and growth of normal tissues [42,43]. *IGF2BP1* regulates these cell functions by binding to target RNAs such as *IGF2*, *c-MYC*, *CD44* and *GLI1* and affecting their translatability, stability or localization [44,45]. *IGF2BP1* is highly expressed in zygotic and embryonic stages, but almost not at all in normal adult cells. Knockdown of *IGF2BP1* may result in significantly smaller body with hypoplastic tissues among almost all organs in mice [46]. It was suggested that *IGF2BP1* may be very important for skeletal muscle development in duck.

Cysteine and glycine rich protein 3 (CSRP3) encodes muscle LIM protein (MLP), which is a member of cysteine-rich protein (CRP) family and is specifically expressed in skeletal muscle and myocardium [47]. MLP is involved in transcriptional regulation, cell fate determination, cell adhesion and movement, cytoskeleton tissue and signal transduction. Its multiple functions have an important influence on the physiology and pathology of the heart and skeletal muscle [48,49]. *MLP* has been shown to bind to transcription factors such as *MyoD* and Myogenin in the nucleus and promote myogenic differentiation [50,51]. *CSRP3* promotes myoblast differentiation. It is first expressed and accumulates in the nucleus when the myotubes form and grow [52]. *CSRP3* silencing leads to down-regulation of myogenic gene expression and up-regulation of atrophy-related genes. In addition, the apoptosis induced by *CSRP*3 silencing is reduced [53]. *CSRP3* may be an interesting candidate gene to explain the phenotypic differences of skeletal muscle development in duck.

Secreted phosphoprotein 1 (SPP1), encoding osteopontin (OPN), is a key inflammatory cytokine involved in tissue remodeling. SPP1 plays an important role in key biological processes such as development, wound healing, and immune response [54]. The role of *SPP1* is to interact with integrins and *CD44* on the cell surface, regulating cell-cell and cell-matrix interactions. *SPP1* can be used as a candidate gene for increasing body weight in sheep breeding [55]. OPN in myoblasts may be involved in regulating myogenesis and inflammation during the early stage of muscle regeneration promoting muscle repair. In vitro, soluble OPN protein promotes the proliferation of C2C12 myoblasts and reduces fusion and migration, while insoluble OPN protein promotes cell adhesion and fusion [56]. Nghiem, P.P. et al. found that *OPN* may interact with AKT1/MSTN/FoxO1 to affect the development of normal and dystrophic muscle [57]. Matsumoto, H. et al. suggested that a non-synonymous mutation of *SPP1* gene affected carcass weight of beef cattle [58]. Therefore, *SPP1* may play an important role in the development of duck skeletal muscle.

Kelch like proteins (KLHLs) are involved in a variety of cellular and biological functions, such as regulation of actin cytoskeleton, cell adhesion, morphological processes, gene expression and cell signaling [59,60]. Kelch-like protein 31 (KLHL31) is a member of Kelch-like gene family. It is well known that *MEF*2 plays an important role in regulating muscle development. Papizan, J.B. et al. found that the skeletal muscle specific gene *KLHL31* can be activated by *MEF2*, and the deletion of *KLHL31* in mice led to retardation of postnatal skeletal muscle growth and degeneration of sarcomere [61]. The expression of *KLHL31* is regulated by myogenic signal and *Myf*-*5* during skeletal myogenesis [62]. Studies have reported that the Wnt signaling pathway plays an important role in embryonic myogenesis and muscle repair, and *KLHL31* regulates embryonic myogenesis by weakening the β-catenin dependent Wnt signaling pathway [63]. Onteru, S.K. et al. indicated that *KLHL31* was associated with loin muscle area in pig [64]. *KLHL31* may be very important in the regulation of skeletal muscle development in duck.

### 4.5. Analysis of GO and KEGG Pathway

To further determine the function of DEGs, functional categorization of all DEGs was performed using GO annotation. The genes *MYL4*, *IGF2BP1*, *CSRP3*, *SPP1*, *KLHL31*, *OPN*, *LAMB2*, *LAMA2*, *ITGB1*, *ACTN1*, *FGF19* and *MAP6*, which are responsible for muscle synthesis or metabolism, were highly enriched in the biological process. According to GO results related to muscle growth at each developmental stage, DEGs were enriched in the category of “myosin complex”, “myofibril” and “proteinaceous extracellular matrix” and were the most important subcategories in cellular components. The most abundant subcategories in molecular function category were “extracellular matrix structural constituent”, “muscle alpha-actinin binding”, and “microtubule motor activity”. The biological processes categories, including “endodermal cell differentiation”, “muscle cell cellular homeostasis”, “skeletal muscle tissue growth” and “skeletal muscle cell differentiation”, were significantly regulated, indicating that DEGs played an important role in regulating duck skeletal muscle. The KEGG analysis of DEGs was also performed, and the pathways including oxidative phosphorylation, ECM-receptor interaction, focal adhesion, carbon metabolism and biosynthesis of amino acids were significantly enriched. In addition, *MYL4*, *IGF2BP1*, *CSRP3, SPP1*, *KLHL31*, *LAMB2*, *LAMA2*, *ITGB1* and *OPN*, which regulate the development of skeletal muscle, were highly enriched in GO terms and signifcantly expressed in KEGG pathways.

The development of embryonic skeletal muscle requires a lot of energy metabolism, and the oxidative phosphorylation is the main source of energy, which exists in most types of muscle fibers in the form of ATP [65]. The energy is provided by oxidative phosphorylation relying on the respiratory and cardiovascular systems, which deliver oxygen, carbohydrate and fat to the contractile skeletal muscle [66]. Lee, H. et al. found that there was a mitochondrial oxidative phosphorylation complex in the cell membrane of skeletal muscle [67]. The main components of ECM are collagen, proteoglycan and adhesion glycoprotein. The specific interaction between cells and ECM is mediated by transmembrane molecules or other cell surface related components, which directly or indirectly control cell adhesion and migration. ECM plays an indispensable role in a variety of cellular responses such as transcription, inflammation, proliferation and differentiation [68]. Focal adhesions (FAs) link matrix-attached transmembrane integrin receptors to actin cytoskeleton via a complex of anchoring proteins. The assembly of FAs and the formation of actin stress fibers are regulated by growth factor-inducing intracellular signals through the activation of GTP-binding protein Rho [69]. Focal adhesion kinase (FAK) plays a regulatory role in skeletal muscle differentiation and participates in insulin signaling and MAPK signaling [70]. Notably, the interaction between FAs and ECM is involved in the regulation of many intracellular pathways of cell movement, proliferation and differentiation [71]. In addition, carbon metabolism and biosynthesis of amino acids also play an important role in skeletal muscle development [72,73]. The results of this study have important implications for predicting the function of new genes and exploring the genetic mechanisms that may play a role in duck muscle growth and development.

### 4.6. Analysis of Protein–Protein Interaction Network

The PPI network showed that *LAMB2*, *LAMA2*, *ITGB1*, *IGF2BP1*, *SPP1*, *KLHL31*, *CSRP3*, and *OPN* were in the network and regulated the duck skeletal muscle development. *LAMB2* and *LAMA2* play an important role in muscle development. Mutations of *LAMA2* and *LAMB2* cause congenital muscular dystrophy (muscle atrophy) in embryos [74,75]. The expression of *ITGB1* in muscle is critical for development of neuromuscular junction. In the absence of *ITGB1* expression in skeletal muscle, the interaction between motoneurons and muscle is defective, which prevents normal presynaptic differentiation [76]. Silencing *ITGB1* and *FAK* inhibits the migration of bovine skeletal muscle-derived satellite cells [77] and Integrin/FAK pathway promotes myoblast differentiation by regulating the expression of *MyoD* and *Cdc42* [78]. Overall, these DEGs may play important roles in muscle growth and development processes in Pekin duck.

## 5. Conclusions

In summary, the transcriptome profiles of breast and leg muscle in Pekin duck at different developmental stages were established using RNA-Seq. The annotations of SNP, InDel and AS in each sample were performed, and 299 novel genes with ≥2 exons were obtained. Subsequent bioinformatic analyses suggested that some DEGs, such as *MYL4*, *IGF2BP1*, *CSRP3*, *SPP1* and *KLHL31*, as well as *LAMB2*, *LAMA2*, *ITGB1* and *OPN*, and pathways such as oxidative phosphorylation, ECM-receptor interaction, focal adhesion, carbon metabolism, and biosynthesis of amino acids, were indispensable for the process of muscle growth and development. The transcriptome of duck skeletal muscle in this study can greatly broaden our understanding of gene expression regulation and network related to skeletal muscle development at different growth stages.

## Figures and Tables

**Table 1:** Primer sequences for qPCR validation and sex-determination.

Groups	Primer Name	Primer Sequence (5′-3′)	Size	Regulated
	*β-actin*	F: CCCTGTATGCCTCTGGTCG	194 bp	
		R: CTCGGCTGTGGTGGTGAAG		
	*GAPDH*	F: CTGGTGCTGAATACGTTGTGG	198 bp	
		R: CTGGTGCTGAATACGTTGTGG		
PE17B_vs_ PE21B	*HOXC8*	F:ACTACTGCTCCCACCTTCCA	168 bp	DOWN
		R:AAGCGATTGCCTCGGTAGAG		
	*KLHL31*	F: AACCAGTGCGTGACAGTGAT	171 bp	UP
		R: GCTGAAGTGGGTACGCTTCT		
PE21B_vs_ PE27B	*DUSP10*	F:GGCAAGATCACCGTCTTGGA	173 bp	UP
		R:TCCTTGCCTTCTCGCTTGAG		
	*FBXO*32	F: GTCGGCAAATCTGTCCTGGT	196 bp	UP
		R: GGCTAACCAGGTCTCTCCCA		
PE27B_vs_ PM6B	*ADGRL3*	F:CCAATGCTCTGCTTCGTCCT	143 bp	DOWN
		R:GGCATTGTTCAGAAGCCCCT		
	*POSTN*	F: GCAGGGAGCTGGAACTGAG	148 bp	DOWN
		R: TGTTGCTCCTCCTTGTGTCC		
PE17L_vs_ PE21L	*MFRP*	F:AGTTCTGCAACCCCGTCTTC	151 bp	DOWN
		R:CAGGTGAACCTACAGTCGGC		
	*GPR*37	F: CGCCAGTCCTCCTTTTCTGT	175 bp	DOWN
		R: ATTTCACGACGGATGGTGCT		
PE21L_vs_ PE27L	*PCSK2*	F:TGTAGCTGAAGCATGGGAGC	128 bp	UP
		R:TGAAGTCGTAGCTGGCTTGG		
	*MYL4*	F:CCTGACCCCAAAAAGGATGC	116 bp	DOWN
		R:AACTCTTCGATCTGCTCGGC		
PE27L_vs_ PM6L	*TDP1*	F:GCTTGGTTCTACCCCTGGAC	132 bp	DOWN
		R:AACTGTCCAACAACAGGCCA		
	*A* *XIN* *2*	F: GCTACCAAGACCTACATAAG	222 bp	DOWN
		R: GAGATAGCCACAGACAACT		

**Table 2:** RNA-Seq data from breast and leg muscle of Pekin duck.

Samples	Clean Reads	Clean Bases	GC Content	Q30 Value
PE17B1	21,975,680	6,554,209,528	51.62%	93.01%
PE17B2	24,902,534	7,429,488,988	50.72%	92.96%
PE17B3	27,675,557	8,261,513,254	51.50%	93.12%
PE17L1	29,364,381	8,772,703,244	51.20%	92.69%
PE17L2	31,153,547	9,309,596,992	51.11%	93.22%
PE17L3	24,302,063	7,263,420,576	50.37%	92.66%
PE21B1	22,936,235	6,848,636,870	51.28%	92.96%
PE21B2	25,545,188	7,617,899,452	51.47%	92.59%
PE21B3	23,616,893	7,047,370,582	50.76%	93.11%
PE21L1	23,419,211	7,007,727,954	51.72%	93.02%
PE21L2	21,134,566	6,316,620,646	51.41%	93.05%
PE21L3	25,503,895	7,612,558,182	51.26%	93.11%
PE27B1	23,860,738	7,133,252,550	50.70%	92.86%
PE27B2	23,257,098	6,947,415,186	50.96%	92.74%
PE27B3	21,883,412	6,534,290,346	51.66%	92.93%
PE27L1	24,872,668	7,420,346,180	51.81%	92.88%
PE27L2	23,784,499	7,108,311,764	51.94%	92.91%
PE27L3	27,447,436	8,197,076,086	51.74%	92.71%
PM6B1	27,026,755	8,072,713,512	53.75%	92.78%
PM6B2	24,555,447	7,338,843,490	53.63%	92.87%
PM6B3	22,180,858	6,629,939,152	54.51%	93.24%
PM6L1	27,342,141	8,173,286,426	53.45%	93.48%
PM6L2	25,702,190	7,668,643,964	53.48%	93.34%
PM6L3	21,228,238	6,343,509,968	53.24%	92.93%

Note: Clean reads: Paired-end numbers of Clean Data; PE17B: Breast muscle of Pekin duck on day 17 of the incubation period; PE17L: Leg muscle of Pekin duck on day 17 of the incubation period.

**Table 3:** The numbers of alternative splicing.

Samples	AS Number	Samples	AS Number
PE17B1	42,619	PE17L1	45,818
PE17B2	43,505	PE17L2	47,889
PE17B3	45,395	PE17L3	47,567
PE21B1	41,937	PE21L1	36,118
PE21B2	42,880	PE21L2	39,346
PE21B3	43,413	PE21L3	44,668
PE27B1	43,374	PE27L1	36,949
PE27B2	45,707	PE27L2	35,841
PE27B3	38,412	PE27L3	37,077
PM6B1	40,585	PM6L1	39,332
PM6B2	35,912	PM6L2	38,184
PM6B3	32,256	PM6L3	36,346

**Table 4:** Number of new genes.

Annotated Databases	New Gene Number
COG	26
GO	97
KEGG	55
KOG	77
Pfam	68
Swiss-Prot	55
eggNOG	154
Nr	297
All	299

**Table 5:** Number of annotated differentially expressed genes (DEGs) in breast and leg muscle of Pekin duck.

DEG Set	Total	COG	GO	KEGG	KOG	NR	Pfam	Swiss-Prot	eggNOG
PE17B_vs_ PE21B	358	123	279	225	247	357	324	263	344
PE21B_vs_ PE27B	1511	495	1206	1021	1070	1503	1354	1090	1446
PE27B_vs_ PM6B	3643	1289	2924	2448	2656	3630	3342	2635	3519
PE17L_vs_ PE21L	624	238	492	417	443	621	561	463	598
PE21L_vs_ PE27L	274	82	207	171	167	274	233	186	258
PE27L_vs_ PM6L	2323	841	1896	1614	1691	2316	2152	1744	2265

## Data Availability

The datasets generated for this study can be found in the NCBI SRA. The Submission of Pekin duck: Submission: SUB8203594 and SUB8204071, Bioproject #PRJNA665336 and Biosamples #SAMN16251841-SAMN16251852 (Breast muscle); Bioproject #PRJNA665337 and Biosamples #SAMN16251854-SAMN16251865 (Leg muscle).

## Supplementary Materials

The following are available online at https://www.mdpi.com/2076-2615/11/3/834/s1, Figures S1–S3 PPI of DEGs in breast muscle of Pekin duck at different time periods (PE17B_vs_PE21B; PE21B_vs_PE27B, and PE27B_vs_PM6B). Figures S4–S6 PPI of DEGs in leg muscle of Pekin duck at different time periods (PE17L_vs_PE21L; PE21L_vs_PE27L, and PE27L_vs_PM6L). Table S1. The concentration and RIN value of sample RNA. Table S2. The most enriched cellular components of GO terms related to muscle development. Table S3. The most enriched molecular function of GO terms related to muscle development. Table S4. The most enriched biological process of GO terms related to muscle development. Table S5. Alternative splicing events of some skeletal muscle related genes. Table S6. The most enriched cellular components related to muscle development. Table S7. The most enriched molecular function of GO terms related to muscle development. Table S8. The most enriched biological process of GO terms related to muscle development. All authors have read and agreed to the published version of the manuscript.
