# Supplementary material for: Characterization and Comparative Transcriptomic Analysis of Skeletal Muscle in Pekin Duck at Different Growth Stages Using RNA-Seq
Source: Animals (Basel). 2021 Mar 16;11(3):834. doi: 10.3390/ani11030834 (PMC8000258; doi:10.3390/ani11030834)
Supplement: Supplementary file 1 [file animals-11-00834-s001.pdf]

PE17B1_PE17B2_PE17B3_vs_PE21B1_PE21B2_PE21B3.ppi.cytoscapeInp

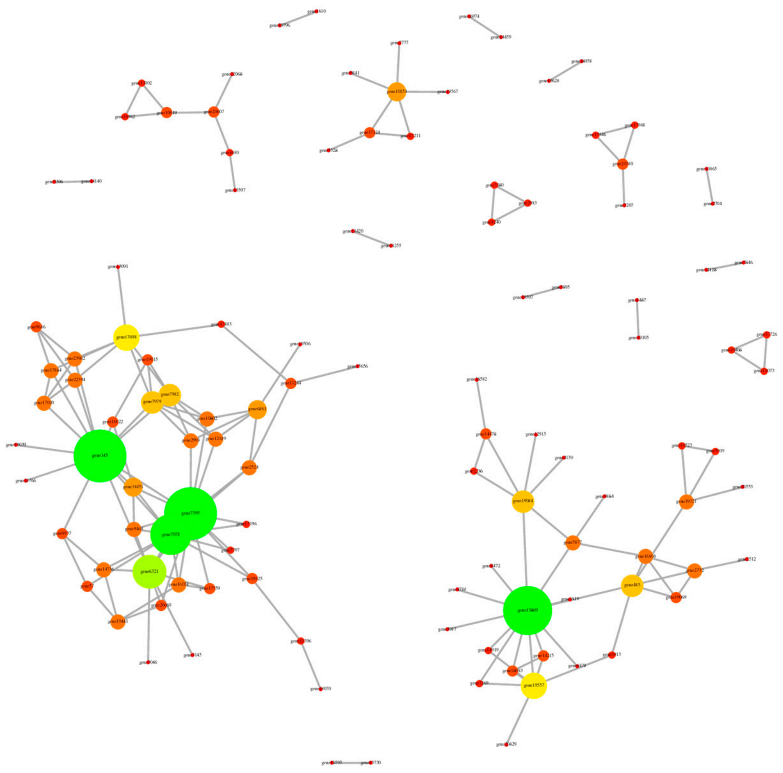

Figure S1. PPI of DEGs in breast muscle of Pekin duck at different time periods (PE17B_vs_PE21B; PE21B_vs_PE27B, and PE27B_vs_PM6B).

PE21B1_PE21B2_PE21B3_vs_PE27B1_PE27B2_PE27B3.ppi.cytoscapeInp

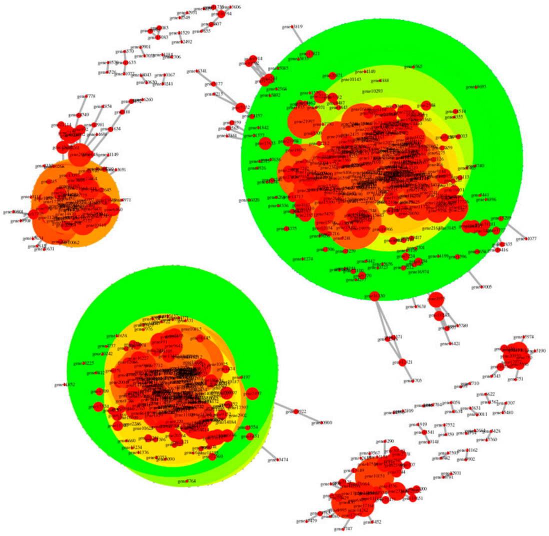

Figure S2.PPI of DEGs in breast muscle of Pekin duck at different time periods (PE17B_vs_PE21B; PE21B_vs_PE27B, and PE27B_vs_PM6B).

PE27B1_PE27B2_PE27B3_vs_PM6B1_PM6B2_PM6B3.ppi.cytoscapeInpu

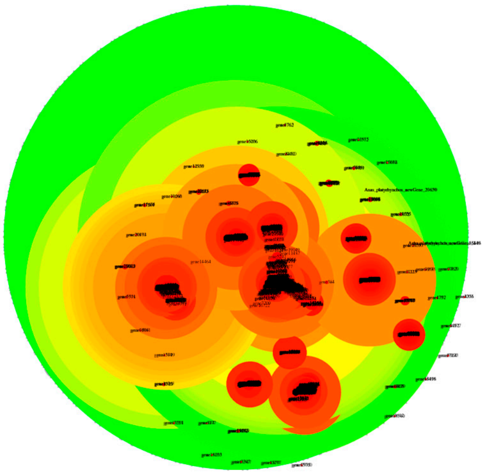

Figure S3.PPI of DEGs in breast muscle of Pekin duck at different time periods (PE17B_vs_PE21B; PE21B_vs_PE27B, and PE27B_vs_PM6B).

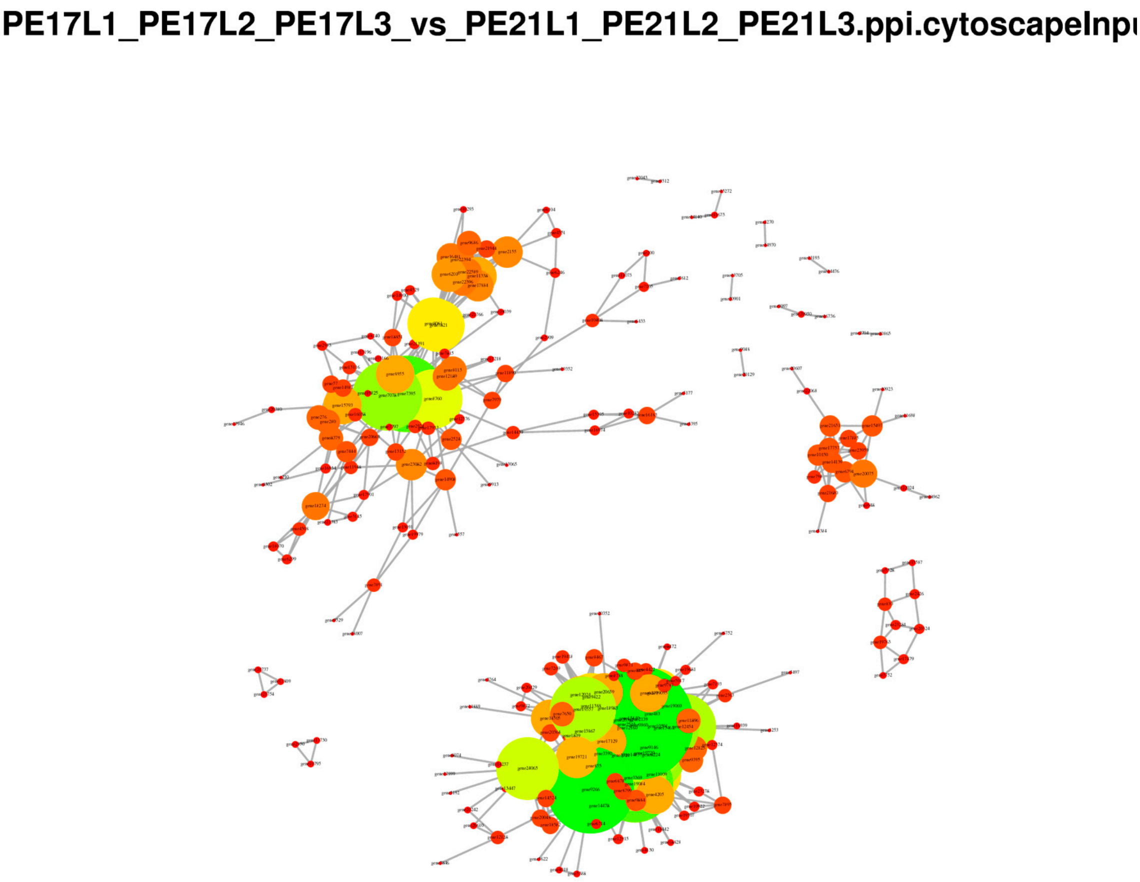

Figure S4.PPI of DEGs in leg muscle of Pekin duck at different time periods (PE17L_vs_PE21L; PE21L_vs_PE27L, and PE27L_vs_PM6L)

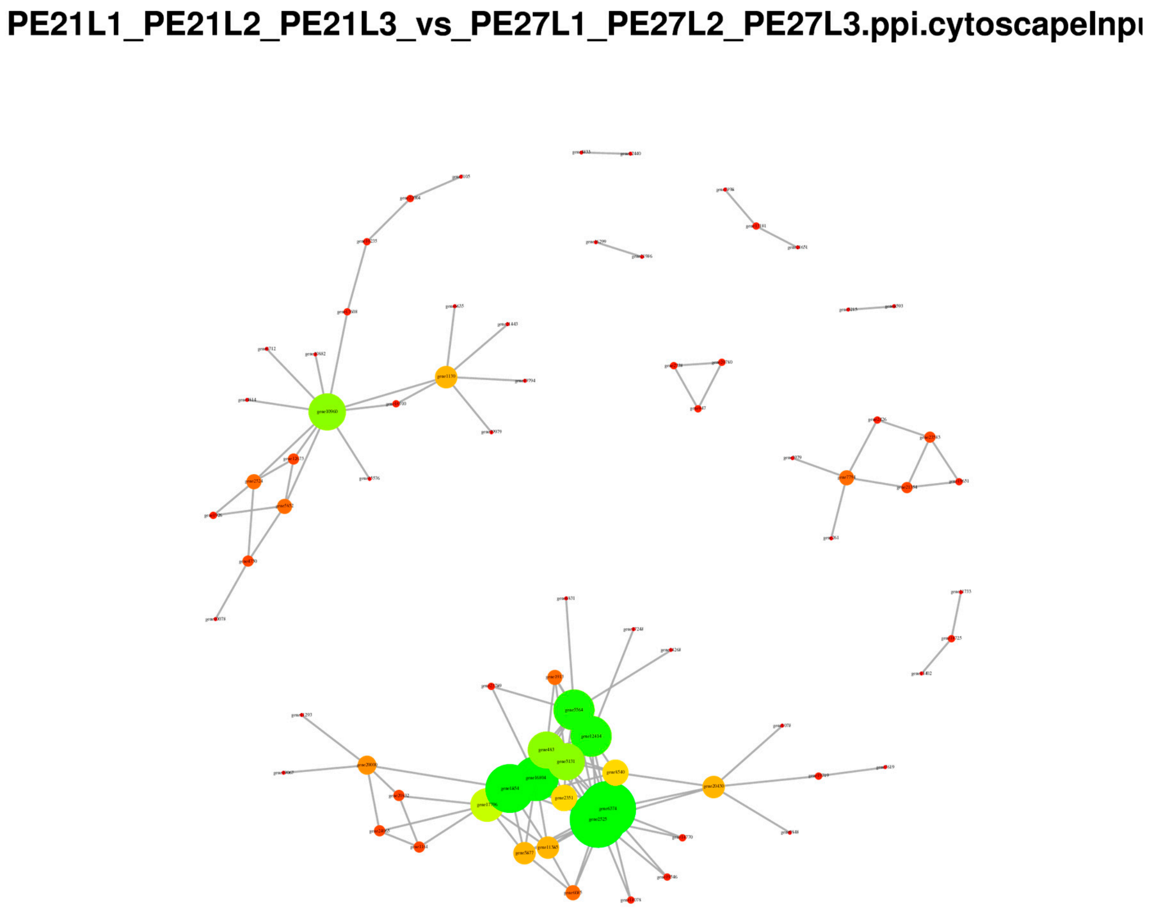

Figure S5.PPI of DEGs in leg muscle of Pekin duck at different time periods (PE17L_vs_PE21L; PE21L_vs_PE27L, and PE27L_vs_PM6L)

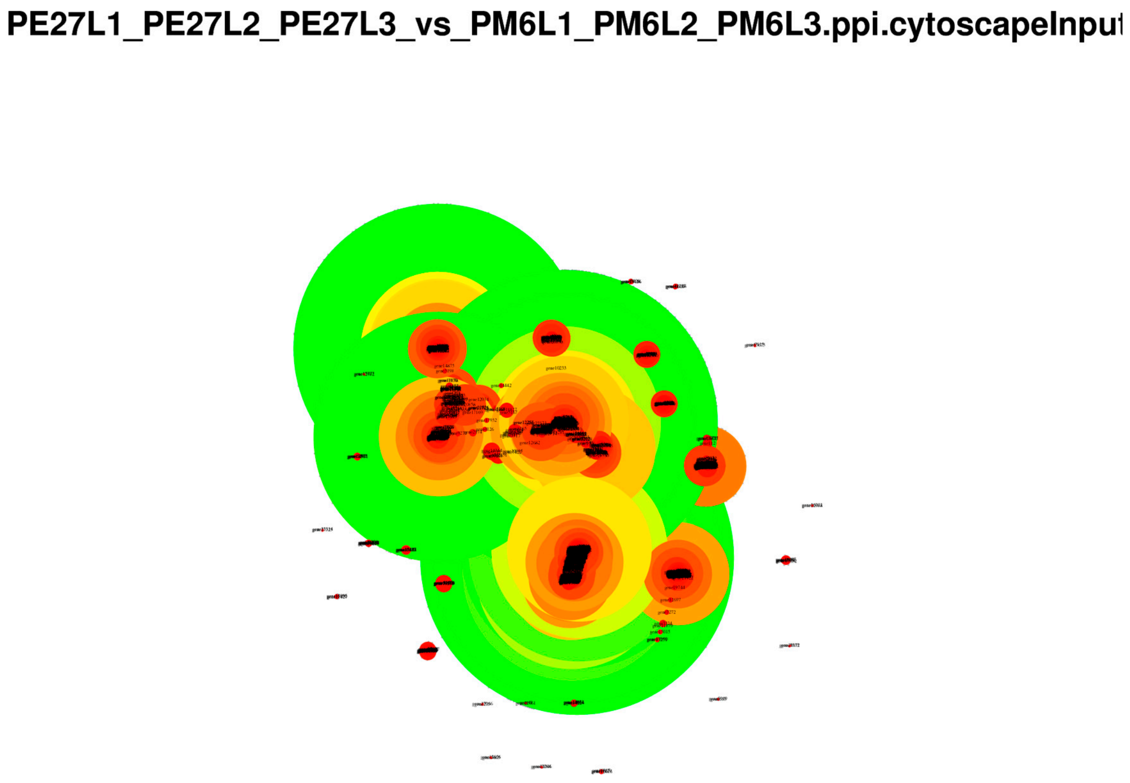

Figure S6. PPI of DEGs in leg muscle of Pekin duck at different time periods (PE17L_vs_PE21L; PE21L_vs_PE27L, and PE27L_vs_PM6L)

Table S1. The feed composition for Pekin duck.

Ingredient	Content (%)	Nutrient	Content (%)
Corn	56.00	Crude protein	15.700
Soybean meal	23.80	Calcium	0.900
Corn gluten meal	10.00	Total phosphorus	0.680
Limestone	7.00	Available phosphorus	0.450
$CaHPO_4$	1.50	Salt	0.370
Premix	1.00	Lysine	0.760
NaCl	0.30	Methionine	0.387
Lys·HCl	0.30	Methionine + Cystine	0.654
DL-Met	0.10	Isoleucine	0.534
Total	100.00	Threonine	0.579

Tryptophan	0.194
Crude fiber	4.100
Crude fat	3.400
Crude ash	5.200
Avian metabolizable energy	2875 $Mcal\cdot kg^{-1}$

Note: Supplied per kilogram of total diet: Cu ($CuSO_4 \cdot 5H_2O$), 8 mg; Fe ($FeSO_4 \cdot 7H_2O$), 52 mg; Zn (ZnO), 60 mg; Mn ($MnSO_4 \cdot H_2O$), 80 mg; Se ($NaSeO_3$), 0.3 mg; I (KI), 0.2 mg; choline chloride, 1,000 mg; vitamin A (retinyl acetate), 10,000 IU; vitamin D3 (Cholcalciferol), 3,000 IU; vitamin E (DL-α-tocopheryl acetate), 20 IU; vitamin K3 (menadione sodium bisulfate), 2 mg; thiamin (thiamin mononitrate), 2 mg; riboflavin, 10 mg; pyridoxine hydrochloride, 4 mg; cobalamin, 0.02 mg; calcium-D-pantothenate, 20 mg; nicotinic acid, 50 mg; folic acid, 1 mg; biotin, 0.2 mg.

Table S2. The concentration and RIN value of sample RNA.

Breast Muscle	Concentration (ng/μL)	RIN value	Leg Muscle	Concentration (ng/μL)	RIN value
PE17B1	1,517.3	9.2	PE17L1	707.9	8.8
PE17B2	703.4	9.5	PE17L2	456.1	8.8
PE17B3	588.3	7.7	PE17L3	1,179.6	8.9
PE21B1	368.3	8.6	PE21L1	127.3	8.1
PE21B2	341.1	8.7	PE21L2	688.4	8.8
PE21B3	1,848.3	9.2	PE21L3	747.1	9.0
PE27B1	748.9	8.2	PE27L1	690.1	8.5
PE27B2	721.3	8.0	PE27L2	433.7	7.9
PE27B3	1,221.8	8.5	PE27L3	1,880.3	8.6
PM6B1	571.1	8.8	PM6L1	577.2	8.8
PM6B2	538.0	8.5	PM6L2	474.7	8.7
PM6B3	407.5	7.6	PM6L3	486.1	7.8

Table S3. Summary of the sequencing read alignment to the *Anas platyrhynchos* genome**.**

Samples	Total Reads	Mapped Reads	Uniq Mapped Reads	Multiple Map Reads	Reads Map to '+'	Reads Map to '-'
PE17B1	43,951,360	35,639,462 (81.09%)	30,749,646 (69.96%)	4,889,816 (11.13%)	13,990,249 (31.83%)	16,562,659 (37.68%)
PE17B2	49,805,068	40,993,328 (82.31%)	35,716,979 (71.71%)	5,276,349 (10.59%)	16,473,003 (33.07%)	19,177,925 (38.51%)
PE17B3	55,351,114	44,311,118 (80.05%)	39,132,150 (70.70%)	5,178,968 (9.36%)	18,586,324 (33.58%)	20,966,706 (37.88%)

PE17L1	58,728,762	47,524,861 (80.92%)	42,963,437 (73.16%)	4,561,424 (7.77%)	21,140,503 (36.00%)	22,909,148 (39.01%)
PE17L2	62,307,094	51,052,098 (81.94%)	45,171,228 (72.50%)	5,880,870 (9.44%)	21,529,190 (34.55%)	24,209,865 (38.86%)
PE17L3	48,604,126	39,952,088 (82.20%)	35,872,270 (73.80%)	4,079,818 (8.39%)	17,166,510 (35.32%)	19,058,044 (39.21%)
PE21B1	45,872,470	35,846,941 (78.14%)	32,125,528 (70.03%)	3,721,413 (8.11%)	15,877,784 (34.61%)	17,262,584 (37.63%)
PE21B2	51,090,376	40,039,766 (78.37%)	34,745,989 (68.01%)	5,293,777 (10.36%)	16,204,760 (31.72%)	18,751,738 (36.70%)
PE21B3	47,233,786	39,125,043 (82.83%)	34,418,096 (72.87%)	4,706,947 (9.97%)	16,159,632 (34.21%)	18,434,167 (39.03%)
PE21L1	46,838,422	37,104,544 (79.22%)	32,274,663 (68.91%)	4,829,881 (10.31%)	15,189,684 (32.43%)	17,475,416 (37.31%)
PE21L2	42,269,132	33,175,658 (78.49%)	28,289,086 (66.93%)	4,886,572 (11.56%)	12,814,743 (30.32%)	15,351,088 (36.32%)
PE21L3	51,007,790	41,591,051 (81.54%)	35,996,297 (70.57%)	5,594,754 (10.97%)	16,682,082 (32.70%)	19,448,985 (38.13%)
PE27B1	47,721,476	38,711,566 (81.12%)	33,742,148 (70.71%)	4,969,418 (10.41%)	15,560,343 (32.61%)	18,131,049 (37.99%)
PE27B2	46,514,196	37,226,509 (80.03%)	32,640,127 (70.17%)	4,586,382 (9.86%)	15,572,568 (33.48%)	17,638,016 (37.92%)
PE27B3	43,766,824	35,678,279 (81.52%)	30,968,073 (70.76%)	4,710,206 (10.76%)	14,349,622 (32.79%)	16,789,571 (38.36%)
PE27L1	49,745,336	31,111,384 (62.54%)	29,395,650 (59.09%)	1,715,734 (3.45%)	15,453,317 (31.06%)	15,590,358 (31.34%)
PE27L2	47,568,998	38,971,216 (81.93%)	29,341,562 (61.68%)	9,629,654 (20.24%)	9,993,587 (21.01%)	16,431,147 (34.54%)
PE27L3	54,894,872	34,249,110 (62.39%)	32,207,350 (58.67%)	2,041,760 (3.72%)	17,010,093 (30.99%)	17,164,612 (31.27%)
PM6B1	54,053,510	40,164,848 (74.31%)	31,078,844 (57.50%)	9,086,004 (16.81%)	12,420,630 (22.98%)	17,564,493 (32.49%)
PM6B2	49,110,894	35,827,011 (72.95%)	27,179,491 (55.34%)	8,647,520 (17.61%)	10,542,571 (21.47%)	15,546,343 (31.66%)
PM6B3	44,361,716	33,549,073 (75.63%)	25,355,459 (57.16%)	8,193,614 (18.47%)	10,898,666 (24.57%)	14,901,195 (33.59%)
PM6L1	54,684,282	42,675,929 (78.04%)	34,705,013 (63.46%)	7,970,916 (14.58%)	15,369,431 (28.11%)	19,329,123 (35.35%)
PM6L2	51,404,380	40,034,712 (77.88%)	31,436,850 (61.16%)	8,597,862 (16.73%)	12,944,339 (25.18%)	17,670,613 (34.38%)
PM6L3	42,456,476	33,743,381 (79.48%)	26,845,998 (63.23%)	6,897,383 (16.25%)	10,888,983 (25.65%)	14,862,123 (35.01%)

Note: Total Reads: Reads number of Clean Data, not paired-end reads; **Mapped Reads:** Reads Number Mapped to the reference genome and the percentage in Clean Reads; **Unique Mapped Reads:** Reads Number Mapped uniquely mapped to the reference genome and the percentage in Clean Reads; **Multiple Mapped Reads:** Reads number multiplely mapped to reference genome and the percentage in Clean Reads; **Reads Map to '+':** Reads number mapped to the sense chain and the percentage in Clean Reads. **Reads Map to '-':** Reads number mapped to the antisense chain and the percentage in Clean Reads.

Table S4. SNPs from breast and leg muscle of Pekin duck.

Sample	SNP Number	Genic SNP	Intergenic SNP	Transition	Transversion	Heterozygosity
PE17B1	95,694	86,192	9,502	74.28%	25.72%	38.64%
PE17B2	96,583	86,174	10,409	74.16%	25.84%	38.38%
PE17B3	108,584	96,476	12,108	73.96%	26.04%	35.11%
PE17L1	118,248	105,658	12,590	73.75%	26.25%	39.05%
PE17L2	115,941	103,327	12,614	73.71%	26.29%	37.86%
PE17L3	102,949	92,617	10,332	73.99%	26.01%	40.61%
PE21B1	87,751	78,293	9,458	74.18%	25.82%	39.90%
PE21B2	90,999	80,177	10,822	74.00%	26.00%	37.94%
PE21B3	94,759	85,563	9,196	74.17%	25.83%	37.48%
PE21L1	64,295	57,351	6,944	75.03%	24.97%	41.99%
PE21L2	76,639	67,905	8,734	74.51%	25.49%	38.10%
PE21L3	107,827	96,923	10,904	73.99%	26.01%	36.16%
PE27B1	93,580	84,532	9,048	73.84%	26.16%	44.64%
PE27B2	110,719	99,794	10,925	73.56%	26.44%	37.99%
PE27B3	68,744	62,099	6,645	75.03%	24.97%	43.05%
PE27L1	419,968	386,872	33,096	72.34%	27.66%	5.74%
PE27L2	64,582	58,823	5,759	74.90%	25.10%	41.91%
PE27L3	427,493	392,907	34,586	72.33%	27.67%	5.38%
PM6B1	88,384	80,444	7,940	74.55%	25.45%	42.69%
PM6B2	66,061	60,575	5,486	75.50%	24.50%	41.92%
PM6B3	70,099	63,745	6,354	75.24%	24.76%	39.08%
PM6L1	88,895	81,180	7,715	74.71%	25.29%	39.01%
PM6L2	82,870	75,914	6,956	74.86%	25.14%	38.47%
PM6L3	82,631	73,406	9,225	74.85%	25.15%	39.07%

Table S5. Alternative splicing events of some skeletal muscle related genes

gene	event_type	chromosome	event_start	event_end	event_pattern	strand
MYL4	TSS	NC_040073.1	2615571	2615757	2615757	+
MYL4	TSS	NC_040073.1	2616675	2617694	2617694	+

MYL4	TTS	NC_040073.1	2620857	2621110	2620857	+
IGF2BP1	TSS	NC_040073.1	5209001	5209080	5209080	+
IGF2BP1	TSS	NC_040073.1	5209285	5209360	5209360	+
IGF2BP1	TTS	NC_040073.1	5238434	5248351	5238434	+
SPP1	TSS	NC_040073.1	45355344	45355489	45355489	+
SPP1	TTS	NC_040073.1	45358650	45359297	45358650	+
KLHL31	TSS	NC_040073.1	93491968	93492171	93492171	+
KLHL31	TTS	NC_040073.1	93497553	93504157	93497553	+
MyoG	TSS	NC_040073.1	3190297	3190776	3190297	+
MyoG	TTS	NC_040073.1	3187859	3197980	3197980	+
MEF2A	TSS	NC_040073.1	3809967	3810155	3809967	-
MEF2A	TSS	NC_040073.1	3810579	3810958	3810579	-
MEF2A	TSS	NC_040073.1	3814519	3814623	3814519	-
MEF2A	TTS	NC_040073.1	3725648	3729784	3729784	-
MEF2A	SKIP_ON	NC_040073.1	3739093	3739116	3735535,3739093-3739116,3740687	-
MEF2A	SKIP_OFF	NC_040073.1	3739093	3739116	37,355,353,740,687	-
MEF2A	SKIP_ON	NC_040073.1	3754697	3754828	3754631,3754697-3754828,3771129	-
MEF2A	SKIP_OFF	NC_040073.1	3754697	3754828	37,546,313,771,129	-
MEF2A	SKIP_ON	NC_040073.1	3754494	3754631	3751447,3754494-	-

MEF2A	SKIP_OFF	NC_040073.1	3754494	3754631	3754631,3754697<br>37,514,473,754,697	-
MSTN	TSS	NC_040073.1	8408607	8409113	8409113	+
MSTN	TTS	NC_040073.1	8414684	8416472	841468	+

Note: event_start: the event start position; **event_end:** the event end position; **event_pattern:** AS event pattern; **strand:** the chain of gene.

Table S6. The most enriched cellular components related to muscle development.

Comparison group	The most enriched cellular components of GO terms related to muscle development				
PE17B_vs_PE21B	myofibril	myosin complex	contractile fiber part	muscle tendon junction	myofilament
PE21B_vs_PE27B	myofibril	focal adhesion	myosin complex	muscle tendon junction	actin filament bundle
PE27B_vs_PM6B	proteinaceous extracellular matrix	focal adhesion	myosin complex	myofibril	MHC class I protein complex
PE17L_vs_PE21L	myosin complex	myofibril	muscle tendon junction	myofilament	striated muscle thin filament
PE21L_vs_PE27L	myosin complex	muscle tendon junction	proteinaceous extracellular matrix	cell surface	cell junction
PE27L_vs_PM6L	proteinaceous extracellular matrix	MHC class I protein complex	microfibril	focal adhesion	myosin complex

Table S7. The most enriched molecular function of GO terms related to muscle development.

Comparison group	The most enriched molecular function related to muscle development

PE17B_vs_PE21B	extracellular matrix structural constituent	muscle alpha-actinin binding	microtubule motor activity	protein serine/threonine kinase activator activity	protein kinase binding
PE21B_vs_PE27B	microtubule motor activity	microtubule binding	extracellular matrix structural constituent	muscle alpha-actinin binding	structural constituent of muscle
PE27B_vs_PM6B	extracellular matrix structural constituent	motor activity	structural constituent of muscle	muscle alpha-actinin binding	protein kinase activator activity
PE17L_vs_PE21L	extracellular matrix structural constituent	muscle alpha-actinin binding	microtubule motor activity	actin filament binding	fibronectin binding
PE21L_vs_PE27L	microtubule motor activity	muscle alpha-actinin binding	motor activity	protein serine/threonine kinase activator activity	proteasome binding
PE27L_vs_PM6L	extracellular matrix structural constituent	muscle alpha-actinin binding	microtubule motor activity	cytoskeletal protein binding	protein serine/threonine kinase activator activity

Table S8. The most enriched biological process of GO terms related to muscle development.

Comparison group	The most enriched biological process related to muscle development				
PE17B_vs_PE21B	skeletal muscle tissue growth	endodermal cell differentiation	muscle cell cellular homeostasis	regulation of skeletal muscle contraction	embryonic skeletal system morphogenesis
PE21B_vs_PE27B	endodermal cell differentiation	negative regulation of skeletal	skeletal muscle tissue growth	negative regulation of skeletal muscle	muscle contraction

		muscle tissue development		satellite cell proliferation	
PE27B_vs_PM6B	tendon development	skeletal muscle cell differentiation	muscle tissue morphogenesis	regulation of synaptic growth at neuromuscular junction	positive regulation of muscle adaptation
PE17L_vs_PE21L	endodermal cell differentiation	negative regulation of skeletal muscle tissue development	muscle cell cellular homeostasis	regulation of skeletal muscle contraction	muscle organ morphogenesis
PE21L_vs_PE27L	embryonic skeletal system morphogenesis	non-canonical Wnt signaling pathway via JNK cascade	skeletal muscle tissue growth	regulation of molecular function	regulation of Rho protein signal transduction
PE27L_vs_PM6L	tendon development	embryonic body morphogenesis	developmental cell growth	regulation of cell proliferation	regulation of insulin-like growth factor receptor signaling pathway
